# From Resilience Gap to Recovery: A Longitudinal Study of Nurse Job Satisfaction, Seniority, and Managerial Interventions in the Postpandemic Era

**DOI:** 10.1155/jonm/5073265

**Published:** 2026-05-14

**Authors:** Ya-Wen Lee, Fei-Chen Lai, Huei-Jhu Siao, Yi-Chen Huang, Chia-Lan Chen, Yu-Cih Jhuang, Chih-Hao Lin

**Affiliations:** ^1^ Department of Administration, Lutung Christian Hospital, No. 480 Zhongzheng Rd. Lukang Township, Changhua, 505002, Taiwan; ^2^ Graduate Institute of Clinical Nursing, National Chung Hsing University, No. 145 Xingda Rd. South Dist., Taichung, 402202, Taiwan, nchu.edu.tw; ^3^ Department of Nursing, Changhua Christian Hospital, No. 135 Nanxiao St. Changhua City, Changhua, 500209, Taiwan, cch.org.tw; ^4^ College of Health Care, China Medical University, No. 100 Sec. 1 Jingmao Rd. Beitun Dist., Taichung, 406040, Taiwan, cmu.edu.cn; ^5^ Department of Nursing, Hung Kuang University, No. 1018 Sec. 6 Taiwan Blvd. Shalu Dist., Taichung, 433304, Taiwan, hk.edu.tw; ^6^ Big Data and Digital AI Application Center, Changhua Christian Hospital, No. 135 Nanxiao St. Changhua City, Changhua, 500209, Taiwan, cch.org.tw

**Keywords:** COVID-19, job satisfaction, longitudinal study, nurses, Resilience Gap, seniority

## Abstract

**Aim:**

The COVID‐19 pandemic has exerted a profound and enduring impact on nurse job satisfaction. Guided by the Job Demands–Resources (JD–R) model, this study aimed to examine (1) longitudinal differences in job satisfaction trajectories across seniority levels, conceptualized as a “Resilience Gap” driven by dynamic resource depletion, and (2) the emergence of recovery following an adaptive institutional response.

**Design and Methods:**

A 5‐year longitudinal observational design was employed using nine waves of panel data (2020–2025) from 2162 nurses (8009 observations) at a large Taiwanese medical center. Linear mixed‐effects modeling was applied to estimate within‐person changes and between‐group differences over time. In early 2024, nursing leadership implemented a set of generation‐sensitive, hospital‐wide managerial interventions, including structured mentorship, empowerment forums, and autonomous e‐rostering. The 2025 outcomes were interpreted descriptively within a noncausal framework.

**Results:**

From 2020 to 2024, a distinct “Resilience Gap” emerged, with job satisfaction among junior and midcareer nurses declining significantly faster than that of senior nurses. This disparity widened progressively during the prolonged “coexistence phase” of the pandemic. In the final observation period (2025), an observable rebound in job satisfaction was identified among less experienced nurses. This descriptive inflection, while temporally aligned with the implementation of managerial interventions, does not imply causal attribution.

**Conclusion:**

The findings conceptualize resilience as a dynamic regulatory capacity within the JD–R framework, reflecting the ongoing balance between job demands and available resources. The observed recovery suggests that proactive, generation‐sensitive managerial strategies may be associated with mitigating prolonged occupational strain although causal inference cannot be established within the present design.

**Impact:**

This study introduces the “Resilience Gap” as a longitudinal diagnostic construct for workforce monitoring and provides a theoretically grounded, data‐informed foundation for developing adaptive management strategies. The findings highlight the importance of continuous resource alignment, multisource monitoring, and empathetic leadership in sustaining a resilient, multigenerational nursing workforce.

## 1. Introduction

The COVID‐19 pandemic placed unprecedented and sustained pressure on healthcare systems worldwide, with nurses at the forefront of prolonged crisis response [1]. Although declines in nurse job satisfaction have been widely reported [2], emerging evidence suggests that these changes are neither uniform nor static but instead reflect a dynamic process shaped by prolonged exposure to high job demands and unequal access to coping resources across workforce groups.

In the postpandemic era, healthcare systems continue to face critical nursing shortages driven by burnout, early‐career attrition, and declining job satisfaction [3]. Taiwan reflects this global trend, with increasing instability among younger nurses [2]. These challenges highlight the need to move beyond descriptive accounts toward theoretically grounded, longitudinal analyses capable of informing targeted workforce strategies.

A key yet underexplored factor is the multigenerational composition of the nursing workforce. Nurses at different career stages exhibit distinct work values, expectations, and coping capacities, particularly regarding work–life balance and professional development [4, 5]. These differences suggest that the impact of prolonged crises such as COVID‐19 may be unevenly distributed, potentially exacerbating disparities in workforce well‐being.

To address this complexity, this study adopts the Job Demands–Resources (JD–R) model. Within this framework, the pandemic represents a sustained escalation of job demands, including workload intensity, emotional strain, and organizational uncertainty, counterbalanced by job and personal resources such as experience, autonomy, and coping capacity [6, 7]. Extending this framework, resilience is conceptualized not as a fixed trait but as a dynamic regulatory capacity governing the balance between job demands and available resources over time. From this perspective, resilience reflects an ongoing process of resource mobilization and adaptation in response to fluctuating environmental pressures [8].

Building on this theoretical lens, we introduce the concept of the “Resilience Gap,” defined as a longitudinal disparity in job satisfaction trajectories across seniority levels. This gap reflects a process of dynamic resource depletion, whereby sustained job demands progressively exceed the available resource pool—particularly among junior and midcareer nurses who are simultaneously navigating early‐career transitions. In contrast, senior nurses, with greater accumulated resources, may be better positioned to buffer prolonged occupational strain.

Despite increasing attention to nurse well‐being, three key gaps remain. First, most studies rely on cross‐sectional designs, limiting the ability to capture temporal dynamics [9]. Second, few studies extend into the prolonged “coexistence” phase of the pandemic, where chronic stressors may have distinct effects [10]. Third, there is limited longitudinal evidence examining whether targeted managerial interventions can mitigate disparities in workforce well‐being [4].

To address these gaps, this study employs a 5‐year longitudinal design to examine the emergence and evolution of the Resilience Gap and to descriptively assess potential recovery following generation‐sensitive managerial interventions implemented in 2024 [11–13]. By integrating problem identification, theoretical interpretation, and organizational response within a unified framework, this study contributes to the understanding of workforce resilience in complex healthcare environments.

Based on the JD–R framework, the following hypotheses are proposed:

• Hypothesis 1 (The Pandemic Effect): nurse job satisfaction will decline over time due to sustained high job demands.
• Hypothesis 2 (The Resilience Gap): seniority, as a proxy for accumulated job resources, will buffer stress effects; thus, junior and midcareer nurses will experience steeper declines in job satisfaction.
• Hypothesis 3 (The Prolonged Crisis Effect): the Resilience Gap will widen during the prolonged “coexistence phase” compared with the initial acute phase.
• Hypothesis 4 (Adaptive Response and Emerging Recovery): following the 2024 interventions, job satisfaction among junior and midcareer nurses will show an observable rebound, interpreted descriptively within a noncausal framework.

## 2. Materials and Methods

### 2.1. Research Design

This study employed a 5‐year longitudinal observational design using secondary panel data (2020–2025) to examine dynamic trajectories of nurse job satisfaction. The analytical framework was grounded in growth curve modeling within a linear mixed‐effects modeling (LMM) approach, enabling the simultaneous estimation of within‐person change and between‐person heterogeneity over time.

The COVID‐19 pandemic was conceptualized as a naturally occurring, prolonged macrolevel stressor within the JD–R framework. To capture its evolving impact, an event impact analytic structure was incorporated to compare two predefined temporal phases: the initial “severe phase” and the subsequent “coexistence phase.” This approach was specifically designed to examine whether disparities in job satisfaction trajectories—conceptualized as the “Resilience Gap”—widened as the crisis transitioned from an acute to a chronic condition.

Importantly, this study did not adopt an experimental or quasiexperimental design. In early 2024, a set of hospital‐wide managerial interventions was implemented as part of an adaptive institutional response. Because these interventions were applied universally and not manipulated under controlled conditions, no distinct intervention or control groups were defined, and the study does not attempt to estimate causal intervention effects. Accordingly, the final observation wave (Q3 2025) was treated as a postintervention observational endpoint.

Any observed changes following the 2024 interventions were interpreted descriptively as temporal associations rather than causal effects. This analytical stance was adopted to explicitly account for potential confounding influences, including concurrent postpandemic normalization of healthcare systems.

The reporting of this study adhered to the Strengthening the Reporting of Observational Studies in Epidemiology (STROBE) guidelines (see Supporting Table S1).

### 2.2. Study Population, Setting, and Response Rates

The study was conducted at a large, 130‐year‐old faith‐based medical center in central Taiwan. The institution comprises multiple clinical divisions, including the Main Hospital, Chunghwa District, and Children’s Hospital District, providing comprehensive healthcare services across acute, critical, and community care settings. The main campus employs approximately 5000 staff members and contains 1690 beds.

To ensure sample homogeneity in work intensity and organizational context, the study included only full‐time registered nurses working at the main campus facilities. Personnel from regional branch hospitals and unlicensed nursing staff were excluded.

A census sampling approach was employed. All eligible full‐time registered nurses (approximately 2000 per wave) were invited to participate in nine waves of an online institutional survey conducted between June 2020 and September 2025. A total of 17,514 invitations were distributed, yielding 8009 valid responses (overall response rate: 45.7%). Response rates across waves ranged from 39.8% to 59.0% (see Table 1).

**TABLE 1:** Survey waves, response rates, and descriptive statistics for nurse job satisfaction (2020–2025).

Year & quarter	Surveys distributed	Surveys responded	Response rate	Average satisfaction (standard deviation)
2020 Q2	1954	1056	54.0%	3.66 (0.47)
2020 Q3	1961	976	49.8%	3.71 (0.50)
2020 Q4	1953	778	39.8%	3.69 (0.52)
2021 Q1	1939	795	41.0%	3.67 (0.50)
2021 Q2	1948	789	40.5%	3.70 (0.53)
2022 Q3	1950	834	42.8%	3.62 (0.53)
2023 Q3	1907	1126	59.0%	3.59 (0.52)
2024 Q3	1921	764	39.8%	3.62 (0.55)
2025 Q3	1981	891	45.0%	3.62 (0.53)
Total	17,514	8009	45.7%	3.66 (0.52)

*Note:* The survey timeline can be conceptually divided into two main pandemic phases evaluated in the event impact model: the “severe phase” (2020 Q2–2021 Q2) and the “coexistence phase” (2022 Q3–2025 Q3). In early 2024, a series of generation‐sensitive managerial interventions were implemented as an adaptive institutional response. Consequently, the final survey wave (2025 Q3) serves as an observational endpoint to descriptively assess potential recovery.

The final analytical dataset consisted of 2162 unique nurses contributing 8009 observations, forming an unbalanced longitudinal panel. Given this structure, LMM was selected as the most appropriate analytical method to accommodate missingness and unequal observation frequencies [14].

### 2.3. Research Instruments

The survey instrument consisted of two sections.

Part 1: Demographic and work‐related characteristics: data were collected on personal and professional attributes, including gender, education level, marital status, job title, and tenure. Clinical seniority was operationalized using the standardized five‐level clinical ladder system endorsed by the Taiwan Nurses Association [15]. This system classifies nurses into levels N–N4 based on experience and competency progression, serving as a proxy for accumulated job resources and professional resilience, defined as follows [16]:

Level N (Novice): entry‐level nurses with less than 1 year of clinical experience.

Level N1: requires at least 1 year of experience and the ability to perform general patient care.

Level N2: requires at least 2 years of experience and active participation in critical care.

Level N3: requires at least 3 years of experience, with expanding responsibilities in teaching and holistic care.

Level N4: requires at least 4 years of experience (typically much longer in practice), predominantly functioning in advanced administrative, educational, or research roles.

Part 2: Job satisfaction: job satisfaction was measured using the validated Taiwanese Hospital Nurses’ Job Satisfaction Scale [17]. The instrument comprises 31 items across five domains: supportive practice environment (9 items), professional autonomy and development (9 items), interpersonal interaction and cooperation (6 items), supervisor leadership (4 items), and workload (3 items). All items were rated on a five‐point Likert scale (1 = *very dissatisfied* to 5 = *very satisfied*), with higher scores indicating greater job satisfaction.

An open‐ended question was included to capture qualitative feedback on non‐nursing administrative burdens. The full questionnaire is provided in Supporting Table S2.

### 2.4. Data Collection

Data collection was embedded within an institutional quality improvement initiative initiated in 2020, aligned with national policies promoting nurse‐friendly practice environments. Surveys were administered across nine waves through a secure internal digital platform.

Aggregated results were periodically disseminated within the organization to enhance transparency and inform management decision‐making. These longitudinal data served as the empirical basis for the establishment of the Nursing‐Friendly Workplace Promotion Committee (NFWPC) in 2024, which subsequently guided the implementation of targeted managerial interventions.

### 2.5. Ethical Considerations

This study was approved by the Institutional Review Board of the participating medical center (IRB no. 250713). The requirement for informed consent was waived due to the use of fully deidentified secondary data.

To ensure confidentiality, all data were anonymized prior to analysis. Identifiable information was replaced with coded identifiers by an independent information technology officer. The final dataset was stored on a secure, password‐protected server accessible only to the research team.

### 2.6. Data Analysis

All analyses were conducted using IBM SPSS Statistics Version 29.0, with statistical significance set at a two‐tailed *α* level of 0.05.

The primary analytical approach utilized LMM within a growth curve framework to examine longitudinal changes in job satisfaction [18]. Time was coded sequentially from 0 (Q2 2020) to 8 (Q3 2025).

A four‐step modeling strategy was implemented. Steps 1–3 were designed to establish baseline trajectories and test Hypotheses 1 and 2 (Pandemic Effect and Resilience Gap), while Step 4 evaluated Hypothesis 3 (Prolonged Crisis Effect).

 Step 1: unconditional means model (null model): an intercept‐only model was estimated to partition variance into within‐person and between‐person components. The intraclass correlation coefficient (ICC) was calculated to justify multilevel modeling.
 Step 2: unconditional growth model: time was introduced as a fixed effect to estimate the average trajectory of job satisfaction. Both intercepts (initial satisfaction at time = 0) and slopes (rate of change) were specified as random effects to capture individual variability.
 Step 3: conditional growth model: time‐invariant predictors (clinical ladder level and gender) were included. Cross‐level interaction terms (time × clinical ladder level) were used to examine whether seniority moderated the rate of change in job satisfaction, operationalizing the “Resilience Gap.”
 Step 4: event impact model: a categorical pandemic phase variable was constructed to distinguish the “severe phase” (2020 Q2–2021 Q2) from the “coexistence phase” (2022 Q3–2025 Q3). Interaction terms (pandemic phase × clinical ladder level) were used to assess whether disparities widened during the prolonged phase of the pandemic.

Importantly, the event impact model was not used to evaluate the effectiveness of the 2024 managerial interventions and was not intended to evaluate intervention‐related effects. Instead, it was designed to isolate temporal pattern differences associated with the evolving pandemic context.

To address Hypothesis 4 (Adaptive Response and Emerging Recovery), observed group‐level trends at the final time point (Q3 2025) were descriptively examined relative to model‐estimated trajectories. This approach enabled identification of potential recovery patterns while avoiding causal overinterpretation, given the observational design and the presence of concurrent external influences such as postpandemic normalization.

## 3. Results

### 3.1. Participant Characteristics and Attrition Analysis

The final longitudinal sample comprised 2162 nurses, contributing a total of 8009 observations across nine survey waves. Baseline demographic characteristics are presented in Table 2. The sample was predominantly female (94.3%), held a bachelor’s degree (85.6%), and was largely unmarried (63.8%). The largest proportion of participants was classified at the N2 clinical ladder level (39.2%).

**TABLE 2:** Baseline demographic characteristics and attrition analysis comparing low‐ and high‐frequency responders (*N* = 2162).

Variable	Number	Percentage (%)	Mean (SD)	Attrition analysis (*p* values)	
Gender				< 0.001	^∗∗∗^
Female	2039	94.3			
Male	123	5.7			
Education level				0.013	^∗^
Diploma or below	210	9.7			
Bachelor’s degree	1851	85.6			
Master’s or above	101	4.7			
Marital status				< 0.001	^∗∗∗^
Unmarried	1379	63.8			
Married	763	35.3			
Divorced/widowed	20	0.9			
Clinical ladder level				< 0.001	^∗∗∗^
N	437	21.5			
N1	313	15.4			
N2	798	39.2			
N3	375	18.4			
N4	113	5.6			
Job title				0.208	
Registered nurse	1942	89.8			
Nurse practitioner	162	7.5			
Nurse	58	2.7			
Hospital district				< 0.001	^∗∗∗^
Main hospital district	1740	80.5			
Chunghwa district	125	5.8			
Children’s hospital district	297	13.7			
Participation in nurse‐to‐patient ratio management				0.036	^∗^
Yes	1459	67.5			
No	703	32.5			
Institutional tenure (years)			9.49 (8.95)	< 0.001	^∗∗∗^
Total nursing tenure (years)			10.69 (9.25)	< 0.001	^∗∗∗^

*Note:* Low‐frequency responders were defined as individuals responding to three or fewer of the nine survey waves. Attrition analysis *p* values were derived from chi‐square tests for categorical variables and independent samples *t*‐tests for continuous variables. High‐frequency responders had significantly longer institutional and total nursing tenure than low‐frequency responders. As discussed in the text, this attrition pattern indicates that the longitudinal sample was weighted toward more stable, senior nurses, thereby yielding a conservative estimation of the Resilience Gap.

^∗^ *p* < 0.05*.*

^∗∗^ *p* < 0.01.

^∗∗∗^ *p* < 0.001.

To assess potential attrition bias, baseline characteristics were compared between high‐frequency responders (> 3 waves) and low‐frequency responders (≤ 3 waves) (Table 2). Significant differences were observed across several variables, most notably institutional tenure and total nursing experience (both *p* < 0.001), with high‐frequency responders demonstrating greater stability and seniority.

Accordingly, the longitudinal sample was moderately weighted toward more experienced nurses. As discussed in Section 4.4, this attrition pattern suggests that the observed estimates of the Resilience Gap are likely conservative.

### 3.2. Longitudinal Trajectories: The Emergence of the “Resilience Gap”

A series of LMMs were fitted to examine longitudinal changes in nurse job satisfaction. The unconditional means model yielded an ICC of 0.675, indicating that a substantial proportion of variance (67.5%) was attributable to between‐person differences, thereby supporting the use of multilevel modeling.

Consistent with Hypothesis 1 (Pandemic Effect), the unconditional growth model (Model 1) revealed a significant negative fixed effect of time (*B* = −0.013, *p* < 0.001), indicating an overall downward trajectory in job satisfaction across the study period. Significant random effects for both intercept and slope further suggested considerable heterogeneity in individual trajectories.

The conditional growth model (Model 2) provided strong support for Hypothesis 2 (Resilience Gap). The interaction between clinical ladder level and Time was statistically significant across multiple levels. Compared with the most senior group (N4), nurses at the N (*B* = −0.037, *p* < 0.001), N1 (*B* = −0.031, *p* < 0.001), N2 (*B* = −0.028, *p* < 0.001), and N3 (*B* = −0.018, *p* < 0.05) levels demonstrated steeper declines in job satisfaction over time. Results for Models 1 and 2 are presented in Table 3.

**TABLE 3:** Hierarchical analysis of the growth model for job satisfaction.

Variables	Model 1: unconditional growth		Model 2: conditional growth	
	Estimate (SE)		Estimate (SE)	
Fixed effects				
Initial status (intercept)				
Intercept	3.713 (0.012)	^∗∗∗^	3.726 (0.062)	^∗∗∗^
Gender (Female vs. Male)			−0.018 (0.050)	
Clinical ladder level (N vs. N4)			0.240 (0.049)	^∗∗∗^
Clinical ladder level (N1 vs. N4)			0.030 (0.048)	
Clinical ladder level (N2 vs. N4)			−0.050 (0.041)	
Clinical ladder level (N3 vs. N4)			−0.057 (0.043)	
Rate of change (time slope)				
Time	−0.013 (0.002)	^∗∗∗^	0.001 (0.011)	
Gender ∗ Time			0.012 (0.009)	
Clinical ladder level (N vs. N4) ∗ Time			−0.037 (0.009)	^∗∗∗^
Clinical ladder level (N1 vs. N4) ∗ Time			−0.031 (0.008)	^∗∗∗^
Clinical ladder level (N2 vs. N4) ∗ Time			−0.028 (0.007)	^∗∗∗^
Clinical ladder level (N3 vs. N4) ∗ Time			−0.018 (0.007)	^∗^
Random effects variance				
Intercept	0.187 (0.009)	^∗∗∗^	0.172 (0.009)	^∗∗∗^
Time slope	0.002 (0.000)	^∗∗∗^	0.002 (0.000)	^∗∗∗^
Intercept‐slope covariance	−0.007 (0.001)	^∗∗∗^	−0.005 (0.001)	^∗∗∗^
Within‐person error	0.091 (0.002)	^∗∗∗^	0.090 (0.002)	^∗∗∗^
Model fit				
−2 log Likelihood	8221.877		8164.654	

*Note:* The reference group for clinical ladder level is N4; the reference group for gender is male. As theoretically proposed, Model 1 confirms the general downward trajectory in satisfaction (Hypothesis 1), while the significant interaction terms in Model 2 provide robust evidence for the “Resilience Gap” among junior and midcareer nurses (Hypothesis 2).

Abbreviation: SE, standard error.

^∗^ *p* < 0.05.

^∗∗^ *p* < 0.01.

^∗∗∗^ *p* < 0.001.

These findings indicate that the observed decline in job satisfaction was not uniform across the workforce. Rather, less experienced nurses exhibited a more pronounced deterioration, consistent with the conceptualization of a widening “Resilience Gap.”

### 3.3. The Event Impact Model: Widening of the Gap

To evaluate Hypothesis 3 (Prolonged Crisis Effect), an event impact model was applied to compare job satisfaction trajectories between the “severe phase” and the “coexistence phase” of the pandemic (Table 4).

**TABLE 4:** Estimates of fixed and random effects for the event impact model.

Parameter	Estimate	SE	*p* value	
Fixed effects				
Intercept	3.715	0.048	< 0.001	^∗∗∗^
Pandemic period (1 vs. 2)	0.033	0.048	0.493	
Gender (Female vs. Male)	0.075	0.040	0.064	
Clinical ladder level (N vs. N4)	−0.010	0.038	0.797	
Clinical ladder level (N1 vs. N4)	−0.192	0.035	< 0.001	^∗∗∗^
Clinical ladder level (N2 vs. N4)	−0.241	0.030	< 0.001	^∗∗∗^
Clinical ladder level (N3 vs. N4)	−0.172	0.029	< 0.001	^∗∗∗^
Pandemic period [1] ∗ Gender (Female)	−0.096	0.040	0.018	^∗^
Pandemic period [1] ∗ Clinical ladder level (N)	0.200	0.044	< 0.001	^∗∗∗^
Pandemic period [1] ∗ Clinical ladder level (N1)	0.178	0.039	< 0.001	^∗∗∗^
Pandemic period [1] ∗ Clinical ladder level (N2)	0.150	0.032	< 0.001	^∗∗∗^
Pandemic period [1] ∗ Clinical ladder level (N3)	0.097	0.033	0.003	^∗∗∗^
Random effects variance				
Intercept	0.159	0.006	< 0.001	^∗∗∗^
Within‐person error (residual)	0.102	0.002	< 0.001	^∗∗∗^
Model fit				
−2 log likelihood = 8271.533				

*Note:* Reference groups were: Phase 2 (coexistence phase) for pandemic phase; Male for gender; and N4 for clinical ladder level. As theoretically proposed, the significant interaction terms between pandemic phase and clinical ladder levels provide robust empirical support for Hypothesis 3, demonstrating that the “Resilience Gap” widened significantly during the prolonged coexistence phase.

Abbreviation: SE, standard error.

^∗^ *p* < 0.05.

^∗∗^ *p* < 0.01.

^∗∗∗^ *p* < 0.001.

The interaction between pandemic phase and clinical ladder level was statistically significant. During the Coexistence Phase (reference category), nurses at the N1 (*B* = −0.192, *p* < 0.001), N2 (*B* = −0.241, *p* < 0.001), and N3 (*B* = −0.172, *p* < 0.001) levels reported lower job satisfaction compared with the N4 group.

The corresponding interaction terms indicated that these disparities were less pronounced during the severe phase, suggesting that the gap between senior and less experienced nurses widened as the pandemic evolved into a prolonged condition.

Taken together, these findings demonstrate that disparities in job satisfaction were not static but expanded over time, supporting the hypothesis that prolonged exposure to sustained job demands disproportionately affected junior and midcareer nurses.

### 3.4. Observational Outcomes: The 2025 Satisfaction Rebound

To address Hypothesis 4 (Adaptive Response and Emerging Recovery), observed trends at the final time point (Q3 2025) were descriptively examined.

**FIGURE 1:**
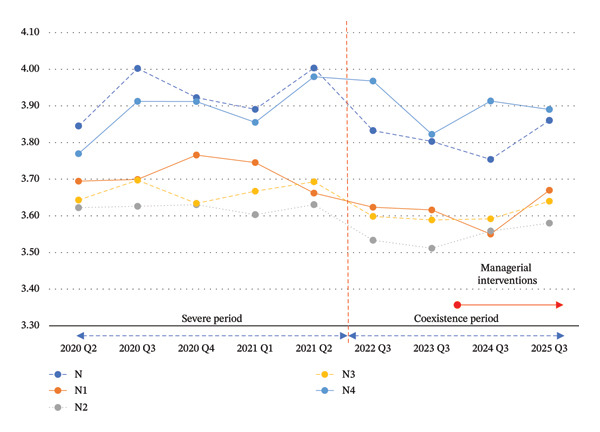
Trajectories of job satisfaction by clinical ladder level (2020–2025). *Note*. The figure displays the mean job satisfaction scores for five clinical ladder levels across nine time points. The dashed vertical line demarcates the “severe phase” and “coexistence phase” of the pandemic. The figure visually illustrates the widening “Resilience Gap” between senior (N4) and other nurses. Notably, the final data point in Q3 2025 shows an observable rebound in satisfaction for junior and mid‐career nurses (N–N3). This positive trend coincides temporally with the adaptive managerial interventions implemented in 2024, providing descriptive evidence of an emerging recovery.

As illustrated in Figure 1, mean job satisfaction scores among junior and midcareer nurses (N–N3 levels) demonstrated a noticeable upward shift following a 4‐year period of decline. In contrast, the trajectory of the most senior group (N4) remained relatively stable.

This observed inflection suggests a potential improvement in job satisfaction among less experienced nurses. However, given the observational nature of the study and the absence of a controlled intervention design, this pattern is interpreted as a temporal association rather than evidence of a causal effect.

The timing of this rebound coincides with the implementation of managerial interventions in 2024, but it may also reflect broader contextual changes, including postpandemic normalization. Therefore, these findings should be interpreted with caution.

## 4. Discussion

This 5‐year longitudinal study provides a comprehensive examination of the evolving trajectories of nurse job satisfaction across the COVID‐19 pandemic and its aftermath. The findings consistently support the four proposed hypotheses, revealing (1) an overall decline in job satisfaction, (2) the emergence of a disparity across seniority levels conceptualized as the “Resilience Gap,” (3) the progressive widening of this gap during the prolonged crisis phase, and (4) an observable rebound among less experienced nurses in the final observation period.

Taken together, these findings suggest that nurse job satisfaction during prolonged systemic stress is not a uniform or static phenomenon but rather reflects a dynamic process shaped by the interaction between job demands and available resources across different workforce groups.

### 4.1. The “Resilience Gap:” Theoretical Interpretation Within the JD–R Framework

The longitudinal findings from 2020 to 2024 provide strong empirical support for the existence of a “Resilience Gap,” characterized by divergent job satisfaction trajectories across clinical ladder levels. While an overall decline in job satisfaction was observed, this decline was substantially steeper among junior and mid‐career nurses compared with their senior counterparts [4].

These patterns can be interpreted within the JD–R framework. The COVID‐19 pandemic can be understood as a sustained escalation of job demands, including increased workload, emotional strain, and organizational uncertainty [7]. Within this context, resilience is conceptualized not as a fixed individual trait but as a dynamic regulatory capacity that reflects the ongoing balance between demands and available resources [8].

From this perspective, the observed Resilience Gap represents a process of differential resource depletion. Senior nurses, with greater accumulated job resources—such as clinical experience, professional autonomy, and adaptive coping strategies—appear better positioned to buffer prolonged occupational strain. In contrast, junior and midcareer nurses, who are simultaneously navigating early‐career transitions, may possess more limited resource reserves, making them more vulnerable to sustained stress exposure [19, 20].

Importantly, these findings extend prior cross‐sectional research by demonstrating that disparities in workforce well‐being are not only present but evolve over time [1, 2]. The Resilience Gap, therefore, can be understood as a longitudinal manifestation of dynamic imbalance within the JD–R system rather than a static difference between groups.

### 4.2. Adaptive Institutional Response and Observed Recovery

Within the JD–R framework, this pattern may be interpreted in relation to changes in the resource environment. In early 2024, a series of hospital‐wide managerial interventions—including structured mentorship, empowerment‐oriented communication mechanisms, and autonomous e‐rostering—were implemented as part of an adaptive institutional response. Rather than being treated as isolated initiatives, these interventions can be understood as resource‐oriented organizational responses that may be particularly relevant for nurses with fewer accumulated professional resources [21, 22].

Specifically, structured mentorship programs may enhance social and professional support, thereby facilitating resource acquisition among early‐career nurses [23–25]. In practice, this program involved a structured mentor–mentee matching process supported by a digital system, with attention to compatibility and workload balance to ensure sustainable mentoring relationships.

Empowerment‐oriented communication platforms, such as participatory committees and executive engagement forums, may strengthen perceived organizational support and psychological empowerment [11]. These mechanisms were operationalized through formal organizational structures, including regular (e.g., periodic or biannual) committee meetings with substantial frontline nurse representation and direct engagement with senior leadership.

Autonomous rostering systems may simultaneously function as a resource enhancer (by increasing job autonomy) and a demand reducer (by alleviating scheduling‐related strain) [26, 27]. In implementation, nurses were allowed to submit scheduling preferences through a structured digital platform, with unit‐level coordination to ensure both flexibility and patient safety requirements.

Collectively, these interventions may be interpreted as modifying the balance between job demands and job resources, particularly for junior and midcareer nurses who were previously more vulnerable to sustained resource depletion. From this perspective, the observed rebound in 2025 may reflect a partial realignment of the JD–R system, in which resource availability became more sufficient to buffer ongoing occupational demands.

However, it is important to emphasize that these interpretations are descriptive rather than causal. Given the observational design and the concurrent influence of broader contextual changes, including postpandemic normalization, the present findings do not permit definitive attribution of the observed recovery to specific interventions. Instead, the JD–R framework provides a theoretically grounded lens through which the temporal co‐occurrence of resource‐oriented organizational changes and improvements in job satisfaction may be interpreted.

Nevertheless, these findings suggest that the Resilience Gap may not be a fixed or irreversible phenomenon, but rather a dynamic condition that can potentially be mitigated through strategic adjustments in the resource environment [28].

### 4.3. Implications for Nursing Management, Education, and Policy

The findings of this study offer several implications for nursing management, education, and health policy.

First, from a managerial perspective, the concept of the Resilience Gap provides a practical diagnostic framework for workforce monitoring. Healthcare organizations may consider routinely tracking longitudinal trends in job satisfaction across clinical ladder levels, rather than relying solely on cross‐sectional indicators. For example, periodic staff surveys combined with administrative data (e.g., turnover rates, sick leave, and patient safety indicators) could be integrated to identify units or subgroups where job demands consistently exceed available resources.

Second, the findings highlight the importance of evaluating not only the implementation but also the efficiency of resource‐oriented interventions. Organizations may consider using pragmatic indicators—such as changes in turnover intention, absenteeism, or retention rates relative to program costs—to assess the cost‐effectiveness of interventions like mentorship programs or flexible scheduling systems. Such evaluations may help prioritize sustainable workforce strategies under resource constraints.

Third, leadership development is central to shaping the resource environment. Nurse managers may function as critical “job resources” by fostering supportive climates, facilitating open communication, and promoting psychological safety [29, 30]. Training programs that incorporate empathetic leadership, active listening, and relational coordination skills may be particularly valuable [31]. Simulation‐based training or reflective practice sessions could be used to strengthen these competencies in real‐world managerial contexts.

At the educational and policy levels, the findings suggest that resilience should be conceptualized as a system‐level capacity rather than solely an individual attribute [32]. Nursing education programs may integrate training on teamwork, adaptive coping, and system awareness to better prepare early‐career nurses for high‐demand environments. From a policy perspective, incorporating workforce well‐being indicators—such as longitudinal job satisfaction trends—into accreditation or quality monitoring systems may support more sustainable workforce planning.

Taken together, these implications suggest that addressing the Resilience Gap requires coordinated efforts across organizational, educational, and policy domains.

### 4.4. Limitations and Future Research

Several limitations should be considered when interpreting these findings.

First, the longitudinal sample was subject to attrition, with high‐frequency responders tending to be more experienced and stable nurses. This pattern introduces potential selection bias. However, sensitivity analyses indicated that individuals who dropped out exhibited steeper declines in job satisfaction, suggesting that the present findings may represent a conservative estimate of the Resilience Gap (Supporting Table S3).

Second, the observed rebound in 2025 occurred within a complex and evolving context. In addition to organizational interventions, broader environmental changes—such as the easing of pandemic‐related pressures—may have influenced job satisfaction. These concurrent factors limit the ability to isolate specific effects within an observational design.

Third, the study was conducted within a single medical center in Taiwan, which may limit the generalizability of specific findings due to contextual factors related to healthcare systems, organizational structures, and cultural norms. In particular, the effectiveness and perceived value of job resources—such as mentorship, empowerment‐oriented communication, and flexible scheduling—may be shaped by locally embedded practices, including hierarchical organizational structures, collective decision‐making processes, and institutional governance models.

Within the JD–R framework, while the overarching mechanism—namely, the dynamic balance between job demands and job resources—is considered broadly applicable across occupational and cultural contexts, the specific forms, accessibility, and effectiveness of job resources are inherently context dependent. For example, empowerment initiatives that rely on direct communication with leadership may function differently in settings with less hierarchical organizational cultures, and flexible scheduling systems may be constrained by workforce policies, staffing ratios, and labor regulations in different healthcare systems.

Accordingly, the observed “Resilience Gap” and its partial recovery should be interpreted within the specific institutional and sociocultural context of Taiwanese healthcare. While the underlying theoretical mechanism of differential resource depletion may be transferable to other settings, the magnitude, trajectory, and responsiveness of such patterns are likely to vary depending on local organizational capacities and policy environments.

Future research should extend longitudinal monitoring beyond the current observation period to examine the sustainability of the observed recovery. Multicenter and cross‐cultural studies are also needed to explore how organizational and policy contexts shape workforce resilience [33]. In addition, qualitative approaches may provide deeper insight into the mechanisms underlying resource depletion and recovery among different nurse groups.

## 5. Conclusion

This 5‐year longitudinal study provides a comprehensive account of the evolving trajectories of nurse job satisfaction across the COVID‐19 pandemic and its aftermath. The findings demonstrate the emergence and progressive widening of a “Resilience Gap,” wherein junior and midcareer nurses experienced a disproportionately greater decline in job satisfaction compared with their senior counterparts.

These results support the conceptualization of resilience as a dynamic, system‐dependent capacity shaped by the ongoing balance between job demands and available resources rather than a fixed individual trait. The identification of the Resilience Gap offers a longitudinal, data‐informed perspective on workforce vulnerability under sustained systemic stress.

In the final observation period, an observable rebound in job satisfaction among less experienced nurses suggests the potential for recovery within this dynamic system. While this pattern coincides temporally with the implementation of organization‐level interventions, it is interpreted descriptively within the constraints of an observational design and may also reflect broader contextual changes.

Overall, the findings indicate that disparities in workforce well‐being are not static but evolve over time and may be associated with changes in the organizational resource environment. Continuous monitoring of workforce dynamics and adaptive, resource‐oriented management strategies may, therefore, be important for supporting a sustainable and resilient nursing workforce in the postpandemic era.

## Author Contributions

Ya‐Wen Lee: conceptualization, resources, supervision, writing–original draft, and writing–review and editing (Lead).

Fei‐Chen Lai: conceptualization, resources, supervision (Supporting), and investigation (Lead).

Huei‐Jhu Siao: conceptualization, investigation, resources, and supervision (Supporting).

Yi‐Chen Huang: data curation, investigation (Supporting), and validation (Lead).

Chia‐Lan Chen: formal analysis, validation, and writing–original draft (Supporting).

Yu‐Cih Jhuang: data curation (Lead), formal analysis, and validation (Supporting).

Chih‐Hao Lin: data curation, writing–original draft, writing–review and editing (Supporting), and formal analysis (Lead).

## Funding

No funding was received for this research.

## Conflicts of Interest

The authors declare no conflicts of interest.

## Supporting Information

Additional supporting information can be found online in the Supporting Information section.

## Supporting information


**Supporting Information 1** Supporting Table S1: STROBE Statement Checklist.

**Supporting Information 2** Supporting Table S2: Items of the Taiwan Hospital Nurses’ Job Satisfaction Scale (2020Q2).

**Supporting Information 3** Supporting Table S3: Sensitivity Analysis for Attrition Bias.

## Data Availability

The data used to support the findings of this study are available from the corresponding author upon reasonable request.
