# Supplementary material for: From Resilience Gap to Recovery: A Longitudinal Study of Nurse Job Satisfaction, Seniority, and Managerial Interventions in the Postpandemic Era
Source: J Nurs Manag. 2026 May 14;2026:5073265. doi: 10.1155/jonm/5073265 (PMC13173754; doi:10.1155/jonm/5073265)
Supplement: Supplementary file 1 — Supporting Information 1 Supporting Table S1: STROBE Statement Checklist. [file JONM-2026-5073265-s001.docx]

Supplementary Table S1：STROBE Statement Checklist

|  | Item No | Recommendation | **Reported?** | **Location** |
| --- | --- | --- | --- | --- |
| **Title and abstract** | 1 | (*a*) Indicate the study’s design with a commonly used term in the title or the abstract | Yes | Title: Page 1 |
|  |  | (*b*) Provide in the abstract an informative and balanced summary of what was done and what was found | Yes | Abstract: Page 2 |
| Introduction | | |  |  |
| Background/rationale | 2 | Explain the scientific background and rationale for the investigation being reported | Yes | Introduction: Page 3 |
| Objectives | 3 | State specific objectives, including any prespecified hypotheses | Yes | Introduction: Page 4 |
| Methods | | |  |  |
| Study design | 4 | Present key elements of study design early in the paper | Yes | Methods 2.1: Page 4 |
| Setting | 5 | Describe the setting, locations, and relevant dates, including periods of recruitment, exposure, follow-up, and data collection | Yes | Methods 2.2: Page 5 |
| Participants | 6 | (*a*) Give the eligibility criteria, and the sources and methods of selection of participants. Describe methods of follow-up | Yes | Methods 2.2: Page 5 |
|  |  | (*b*) For matched studies, give matching criteria and number of exposed and unexposed | N/A | No matched design |
| Variables | 7 | Clearly define all outcomes, exposures, predictors, potential confounders, and effect modifiers. Give diagnostic criteria, if applicable | Yes | Methods 2.3: Page 5 |
| Data sources/ measurement | 8 | For each variable of interest, give sources of data and details of methods of assessment (measurement). Describe comparability of assessment methods if there is more than one group | Yes | Methods 2.3–2.4: Page 5-6 |
| Bias | 9 | Describe any efforts to address potential sources of bias | Yes | Methods 2.2: Page 5  Discussion 4.4: Page 11 |
| Study size | 10 | Explain how the study size was arrived at | Yes | Methods 2.2: Page 5 |
| Quantitative variables | 11 | Explain how quantitative variables were handled in the analyses. If applicable, describe which groupings were chosen and why | Yes | Methods 2.6: Page 6 |
| Statistical methods | 12 | (*a*) Describe all statistical methods, including those used to control for confounding | Yes | Methods 2.6: Page 6 |
|  |  | (*b*) Describe any methods used to examine subgroups and interactions | Yes | Methods 2.6: Page 6 |
|  |  | (*c*) Explain how missing data were addressed | Yes | Methods 2.2: Page 5 |
|  |  | (*d*) If applicable, explain how loss to follow-up was addressed | Yes | Results 3.1: Page 7 |
|  |  | (*e*) Describe any sensitivity analyses | Yes | Discussion 4.4: Page 11  Supplementary Table S3 |
| Results | | |  |  |
| Participants | 13 | (a) Report numbers of individuals at each stage of study—eg numbers potentially eligible, examined for eligibility, confirmed eligible, included in the study, completing follow-up, and analysed | Yes | Results 3.1: Page 7  Table 1: Page 17 |
|  |  | (b) Give reasons for non-participation at each stage | Yes | Methods 2.2: Page 5  Table 1: Page 17 |
|  |  | (c) Consider use of a flow diagram | Partial | No flow diagram, but Table 1 provides wave-by-wave numbers |
| Descriptive data | 14 | (a) Give characteristics of study participants (eg demographic, clinical, social) and information on exposures and potential confounders | Yes | Results 3.1: Page 7  Table 2: Page 18 |
|  |  | (b) Indicate number of participants with missing data for each variable of interest | N/A | No specific missing data counts per variable; LMM handles overall |
|  |  | (c) Summarise follow-up time (eg, average and total amount) | Yes | Methods 2.2: Page 5 |
| Outcome data | 15 | Report numbers of outcome events or summary measures over time | Yes | Table 1: Page 17  Figure 1: Page 22 |
| Main results | 16 | (*a*) Give unadjusted estimates and, if applicable, confounder-adjusted estimates and their precision (eg, 95% confidence interval). Make clear which confounders were adjusted for and why they were included | Yes | Table 3-4: Page 20-21 |
|  |  | (*b*) Report category boundaries when continuous variables were categorized | N/A |  |
|  |  | (*c*) If relevant, consider translating estimates of relative risk into absolute risk for a meaningful time period | N/A |  |
| Other analyses | 17 | Report other analyses done—eg analyses of subgroups and interactions, and sensitivity analyses | Yes | Results 3.3: Page 8 |
| Discussion | | |  |  |
| Key results | 18 | Summarise key results with reference to study objectives | Yes | Discussion 4.1–4.2: Page 9 |
| Limitations | 19 | Discuss limitations of the study, taking into account sources of potential bias or imprecision. Discuss both direction and magnitude of any potential bias | Yes | Discussion 4.4: Page 11 |
| Interpretation | 20 | Give a cautious overall interpretation of results considering objectives, limitations, multiplicity of analyses, results from similar studies, and other relevant evidence | Yes | Discussion 4.2–4.3: Page 9-10 |
| Generalisability | 21 | Discuss the generalisability (external validity) of the study results | Yes | Discussion 4.4: Page 11 |
| Other information | | |  |  |
| Funding | 22 | Give the source of funding and the role of the funders for the present study and, if applicable, for the original study on which the present article is based | Yes | Funding section: Page 13 |

Note: This table was downloaded from the website <https://www.strobe-statement.org/>
