# Supplementary material for: From Resilience Gap to Recovery: A Longitudinal Study of Nurse Job Satisfaction, Seniority, and Managerial Interventions in the Postpandemic Era
Source: J Nurs Manag. 2026 May 14;2026:5073265. doi: 10.1155/jonm/5073265 (PMC13173754; doi:10.1155/jonm/5073265)
Supplement: Supplementary file 2 — Supporting Information 2 Supporting Table S2: Items of the Taiwan Hospital Nurses’ Job Satisfaction Scale (2020Q2). [file JONM-2026-5073265-s002.docx]

**Supplementary Table S2. Items of the Taiwanese Hospital Nurses’ Job Satisfaction Scale (2020 Q2)**

The Taiwanese Hospital Nurses’ Job Satisfaction Scale was originally developed and psychometrically validated by Tzeng et al. [17]. The present study employed the validated Chinese version of the instrument. For the purpose of international dissemination and transparency, an English translation of the items is provided below; this translation has not been independently validated for use in English-speaking populations.

In the context of this study, the scale serves as the primary outcome measure to capture longitudinal changes in nurse job satisfaction within the Job Demands–Resources (JD-R) framework. The five dimensions of the instrument reflect key domains of the work environment that may be conceptually aligned with job resources (e.g., supportive practice environment, professional autonomy, leadership support) and job demands (e.g., workload), thereby enabling a theoretically informed interpretation of changes in satisfaction over time.

The instrument consists of two sections. Section 1 comprises 31 items across five dimensions of job satisfaction. All items are rated on a five-point Likert scale (1 = very dissatisfied to 5 = very satisfied), with higher scores indicating greater perceived job satisfaction. Section 2 collects demographic and work-related characteristics used as covariates in the analysis.

**Section 1. Job Satisfaction Scale**

**Dimension 1. Supportive Practice Environment (9 items)**

1. The personal protective equipment and facilities provided by the institution.
2. The computerization of various work processes.
3. The safety of my work environment.
4. The variety and quantity of medical equipment provided by the institution.
5. The model of patient care implemented by the institution.
6. The institution's support for nurses' continuing education.
7. The professional training provided by the institution.
8. The retirement system of the institution.
9. The opportunities for promotion in my position.

**Dimension 2. Professional Autonomy and Development (9 items)**

1. My current salary and compensation.
2. My level of control over my own work.
3. My ability to handle crisis situations.
4. The degree of professional autonomy in my work.
5. The opportunities I have to participate in important decision-making.
6. The recognition and affirmation I receive from my peers regarding my work competence.
7. The recognition and affirmation I receive from my supervisor regarding my work competence.
8. The sense of achievement I derive from my work.
9. The learning opportunities available to me at work.

**Dimension 3. Interpersonal Interaction and Cooperation (6 items)**

1. My interactions with other healthcare professionals.
2. My collaboration with physicians.
3. My collaboration with other nursing personnel.
4. My interactions with administrative staff.
5. The mutual respect among interdisciplinary team members at work.
6. My interactions with my nursing peers.

**Dimension 4. Supervisor Leadership (4 items)**

1. The support and care I receive from my immediate nursing supervisor.
2. The leadership ability of my immediate nursing supervisor.
3. My immediate nursing supervisor's ability to build team cohesion.
4. The fairness and reasonableness with which my immediate supervisor treats matters (e.g., promotions, evaluations, rewards, and benefits).

**Dimension 5. Nursing Workload (3 items)**

1. The amount of my non-nursing professional workload.
2. The amount of my nursing professional workload.
3. The level of difficulty of my current nursing work.

**Open-ended item:**

Non-nursing professional tasks within the unit that could be improved.

**Section 2. Demographic and Work-Related Information**

1. **Gender**:
   - Female
   - Male
2. **Education Level**:
   - Diploma or below
   - Associate degree / Bachelor's degree
   - Master's degree or above
3. **Marital Status**:
   - Unmarried
   - Married
   - Divorced / Widowed
4. **Clinical Ladder Level**:
   - N
   - N1
   - N2
   - N3
   - N4
5. **Job Title**:
   - Nurse
   - Registered Nurse
   - (Associate) Nurse Practitioner
6. **Institutional Tenure** (in years, including decimals): [Open-ended numerical response]
7. **Total Nursing Tenure** (in years, including decimals): [Open-ended numerical response]
