# Supplementary material for: From Resilience Gap to Recovery: A Longitudinal Study of Nurse Job Satisfaction, Seniority, and Managerial Interventions in the Postpandemic Era
Source: J Nurs Manag. 2026 May 14;2026:5073265. doi: 10.1155/jonm/5073265 (PMC13173754; doi:10.1155/jonm/5073265)
Supplement: Supplementary file 3 — Supporting Information 3 Supporting Table S3: Sensitivity Analysis for Attrition Bias. [file JONM-2026-5073265-s003.docx]

Supplementary Table S3. Sensitivity Analysis for Attrition Bias

1. Analytical Rationale and Approach

To evaluate the robustness of the main findings in the presence of potential attrition bias, a sensitivity analysis was conducted using a pattern-mixture modeling (PMM) approach, which is commonly applied to longitudinal data with non-random missingness.

Participants were stratified into two groups based on response frequency across the nine survey waves: low-frequency responders (≤3 waves) and high-frequency responders (>3 waves). This stratification allows for the examination of whether longitudinal patterns differ between individuals with more limited versus more stable participation.

The conditional growth model (Model 2 in the main analysis) was then estimated separately within each subgroup. This approach was intended to assess the consistency of the observed trajectory patterns rather than to establish causal effects.

2. Results

Supplementary Table S3 presents the results of the PMM-based sensitivity analysis. Across both low- and high-frequency responder groups, the interaction terms between time and lower clinical ladder levels (N, N1, and N2) remained negative, indicating a steeper decline in job satisfaction among less experienced nurses relative to senior nurses (N4).

Although some variations in magnitude were observed between subgroups, the overall pattern of differential decline was directionally consistent across both groups.

3. Interpretation

These findings suggest that the core pattern identified in the main analysis—namely, the presence of a “Resilience Gap” characterized by greater vulnerability among junior nurses—is robust to potential attrition effects.

However, it is important to interpret these results within the broader context of differential response patterns. As noted in the main manuscript, high-frequency responders were more likely to be experienced and stable nurses, whereas individuals with lower participation may include those experiencing greater occupational strain.

Accordingly, while the sensitivity analysis supports the consistency of the observed pattern, the magnitude of the Resilience Gap reported in the main analysis may represent a conservative estimate of the underlying disparity in the full nursing workforce.

Supplementary Table S3 presents the results of the PMM analysis

| Variables | Model 1: Unconditional | | | |  | Model 2: Conditional | | | |
| --- | --- | --- | --- | --- | --- | --- | --- | --- | --- |
|  | low-frequency, <=3 waves | | high-frequency, >3 waves | |  | low-frequency, <=3 waves | | high-frequency, >3 waves |  |
|  | Estimate (SE) |  | Estimate (SE) |  |  | Estimate (SE) |  | Estimate (SE) |  |
| **Fixed Effects** |  |  |  |  |  |  |  |  |  |
| **Intercept** |  |  |  |  |  |  |  |  |  |
| Intercept | 3.743(.021) | *** | 3.701(.015) | *** |  | 3.718(.120) | *** | 3.721(.071) | *** |
| Gender [Female vs. Male] |  |  |  |  |  | .041(.084) |  | -.029(.059) |  |
| Clinical Ladder Level [N vs. N4] |  |  |  |  |  | .203(.099) | * | .222(.055) | *** |
| Clinical Ladder Level [N1 vs. N4] |  |  |  |  |  | -.010(.095) |  | .043(.051) |  |
| Clinical Ladder Level [N2 vs. N4] |  |  |  |  |  | -.120(.091) |  | -.020(.044) |  |
| Clinical Ladder Level [N3 vs. N4] |  |  |  |  |  | -.072(.097) |  | -.038(.045) |  |
| **Time Slope** |  |  |  |  |  |  |  |  |  |
| Time | -0.020(.004) | *** | -0.010(.002) | *** |  | .014(.021) |  | -.006(.012) |  |
| Gender * Time |  |  |  |  |  | -.001(.015) |  | .022(.011) | * |
| Clinical Ladder Level [N vs. N4] * Time |  |  |  |  |  | -.04(.018) | * | -.032(.012) | *** |
| Clinical Ladder Level [N1 vs. N4] * Time |  |  |  |  |  | -.036(.017) | * | -.031(.01) | *** |
| Clinical Ladder Level [N2 vs. N4] * Time |  |  |  |  |  | -.033(.016) | * | -.028(.007) | *** |
| Clinical Ladder Level [N3 vs. N4] * Time |  |  |  |  |  | -.031(.018) |  | -.018(.008) | * |
| **Random Effects Variance** |  |  |  |  |  |  |  |  |  |
| Intercept | .158(.017) | *** | .183(.011) | *** |  | .187(.009) | *** | .172(.010) | *** |
| Time Slope | .000(.000) |  | .002(.000) | *** |  | .000(.000) |  | .002(.000) | *** |
| Intercept-Slope Covariance | .000(.002) |  | -.006(.001) | *** |  | -.001(.001) |  | -.005(.001) | *** |
| Within-Person Error | .131(.006) | *** | .084(.002) | *** |  | .129(.006) | *** | .084(.002) | *** |
| **Model Fit** |  |  |  |  |  |  |  |  |  |
| -2 Log Likelihood | 2890.443 |  | 5252.687 |  |  | 2876.895 |  | 5203.306 |  |
